# Leveraging long read sequencing from a single individual to provide a comprehensive resource for benchmarking variant calling methods

**DOI:** 10.1038/srep14493

**Published:** 2015-09-28

**Authors:** John C. Mu, Pegah Tootoonchi Afshar, Marghoob Mohiyuddin, Xi Chen, Jian Li, Narges Bani Asadi, Mark B. Gerstein, Wing H. Wong, Hugo Y. K. Lam

**Affiliations:** 1 Bina Technologies, Roche Sequencing, Redwood City, CA 94065, USA; 2 Department of Electrical Engineering, Stanford University, Stanford, CA 94305, USA; 3 Department of Statistics, Stanford University, Stanford, CA 94305, USA; 4 Program in Computational Biology and Bioinformatics, Yale University, New Haven, CT 06520, USA; 5 Department of Health Research and Policy, Stanford University, Stanford, CA 94305, USA

## Abstract

A high-confidence, comprehensive human variant set is critical in assessing accuracy of sequencing algorithms, which are crucial in precision medicine based on high-throughput sequencing. Although recent works have attempted to provide such a resource, they still do not encompass all major types of variants including structural variants (SVs). Thus, we leveraged the massive high-quality Sanger sequences from the HuRef genome to construct by far the most comprehensive gold set of a single individual, which was cross validated with deep Illumina sequencing, population datasets, and well-established algorithms. It was a necessary effort to completely reanalyze the HuRef genome as its previously published variants were mostly reported five years ago, suffering from compatibility, organization, and accuracy issues that prevent their direct use in benchmarking. Our extensive analysis and validation resulted in a gold set with high specificity and sensitivity. In contrast to the current gold sets of the NA12878 or HS1011 genomes, our gold set is the first that includes small variants, deletion SVs and insertion SVs up to a hundred thousand base-pairs. We demonstrate the utility of our HuRef gold set to benchmark several published SV detection tools.

Validation of variant calls from high throughput sequencing is crucial to ensure reliability of experiment results and in the development of new methods. Despite recent efforts in generating a validation set from high throughput sequencing data^1^,^2^ and in characterizing complex variation at a population scale^3^, there has been no gold set of variants that covers all size ranges in a single human genome from single nucleotide variants (SNVs) to large structural variants (SVs). We leveraged the whole-genome Sanger sequencing available for the NS12911 genome (HuRef)^4^ to construct the first gold set of variants from a diploid male Caucasian genome that includes variants ranging from one base pair to over one hundred thousand base pairs. Given that Sanger sequencing is the *de facto* standard in validating variant calls and that this is the largest whole-genome set of long Sanger reads available for a single individual thus far, the resulting gold set constructed is comprehensive and of high quality. Additionally, the Sanger sequencing experiments included a number of large BAC libraries, which are able to span large insertions up to 100 K bp. In order to perform cross-platform validation, we sequenced using Illumina HiSeq 2000 (2 × 100 bp) to 140× coverage (a 100× and a 40× set). We also aggregated a number of relevant public datasets and computational approaches to expand our validation.

Although previous works^4^,^5^ have published large sets of variants for the HuRef genome, we found that the accuracy and resolution of these variants were not high enough to be used as validation datasets. During our assessment of these published datasets, we found that some of the reported SVs were imprecise since no read support could be found using junction-mapping for the exact SV breakpoints reported. We also found that a number of small insertions and deletions did not match those called from Illumina sequencing. Closer examination revealed that some of the small insertions and deletions appeared to be sequencing errors in Sanger reads. This can be explained since heterozygous variants are indistinguishable from sequencing errors in low coverage areas. Furthermore, available variant sets for HuRef use hg18/NCBI36 as the reference genome which requires a liftover step to be used with up to date versions of human references and also results in unplaced coordinates and potential incorrect placement. Finally, existing HuRef variant sets are provided in multiple files and formats that need significant processing to be standardized and considered for validation. Our work addresses these issues and provides a usable, comprehensive and accurate set of variants for validation of secondary analysis. We built the gold set with multiple validation approaches using publicly available Sanger sequenced reads from the HuRef project^4^. Part of our validation involved employing computational approaches to the original Sanger reads to refine and extend the existing variant sets. In addition, we also sequenced HuRef using Illumina HiSeq in order to get further cross-platform validation using next generation sequencing. In order to aid accuracy assessment using our gold set, we identified regions of the genome unlikely to have an SV and regions with high-confidence homozygous reference calls for the purpose of identifying false positives. Finally, the HuRef SV gold set was then used to compare the accuracy of several popular SV callers.

We have released all reads and variants to public repositories.

## Material and Methods

### Gold set for structural variants

The Venter genome is unique as it was sequenced to relatively deep (9×) coverage with long Sanger reads and includes several libraries of BAC clones with large insert sizes. This set of reads is very attractive for constructing a high quality set of structural variants. The long reads are able to span the most common classes of repeats (SINE elements) and long insert sizes can resolve large insertions.

**Figure 1:**
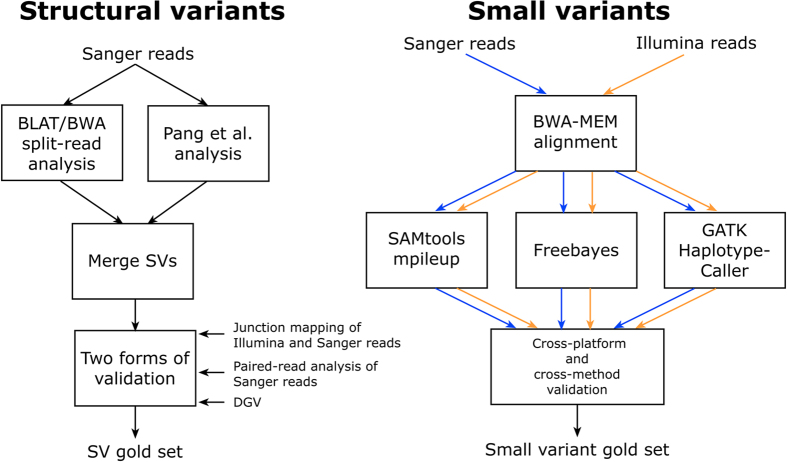
Workflow to construct small variant and SV gold sets.

In order to construct a gold set for structural variants (SV), we first constructed a set of SVs with evidence from the Sanger reads by merging SVs from Pang’s callset^5^ with additional SVs identified from split-read alignments of BWA-MEM (0.7.5a) and BLAT (v34). This set contains SVs that simply have any evidence from Sanger reads and are not stringent enough for validation. In order to construct the gold set these need to be then refined using multiple forms of validation to obtain the gold set SVs (See Fig. 1). This validation included the Database of Genomic Variants (DGV)^6^, junction mapping and discordant read-pair analysis. We used the whole 140× Illumina dataset and all Sanger reads for junction mapping. We also removed known HuRef SVs from DGV before performing the validation. In order to estimate FDR, we used the Sanger reads to construct a set of “no structural variant regions” (NSVR). These are regions where only multiple trimmed Sanger reads uniquely align end-to-end with low edit distance. When estimating the FDR of an SV caller, an NSVR false positive (NSVR-FP) is defined as an SV call that does not match any SV in the gold set and also both breakpoints lie in an NSVR. The combination of the gold set SVs and the NSVR can be used to compare SV calling algorithms. See supplementary for detailed methods.

We further enhanced the comprehensiveness of our SV call set by identifying the tandem repeats (TRs) in HuRef with the Sanger reads. In total we identified 95,852 TRs. These were not used in the gold set construction, but still useful as long Sanger reads can identify tandem repeats that are invisible to short reads. For instance, we successfully identified the previously found uVNTR (upstream variable number of tandem repeats) in HuRef’s MAOA gene.

### Gold set for small variants

In order to construct the HuRef gold set for small variants, we called variants for both Sanger and Illumina (100× set) reads using three state-of-the-art variant callers—GATK’s HaplotypeCaller^7^, FreeBayes^8^ and SAMtools^9^. We followed the recommended practices for each tool when processing. The following criteria were used to identify gold set variants. Firstly, the variant must be called by at least one caller for each platform. This ensures that the sequencing technology-specific biases are minimized for gold set variants. Secondly, the variant must be called by two different variant callers for the datasets. This means that if the same variant was called by only FreeBayes for both the datasets, then this criterion is not satisfied. This criterion would reduce the variant caller specific biases among gold set variants. A variant must satisfy both criteria to be included in the HuRef gold set for small variants (see Fig. 1). This cross-platform and cross-caller approach to generate a gold set is similar to the one taken by the Genome in a Bottle Consortium^1^. It has also been used to improve the quality of variant calls in other settings, such as somatic variants^10^ and structural variants^11^,^12^. Only one criterion needs be satisfied to be included in the complete set. Similar to the NSVRs, we generate reference call regions for small variants by calling reference regions with SAMtools and HaplotypeCaller. The same cross-platform and cross-caller criteria are used to generate the final reference call regions. See supplementary for detailed methods.

### Data Availability. 

Illumina sequences released on SRA as BioProject PRJNA281509. Variants available at http://bioinform.github.io/huref-gs/ and also on the Genome in a Bottle FTP site at ftp://ftp-trace.ncbi.nlm.nih.gov/giab/ftp/technical.

## Results

### Analysis of structural variant gold set

**Figure 2:**
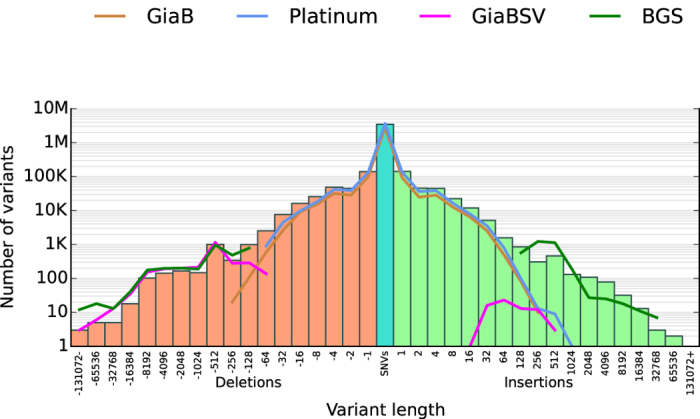
Histogram of size ranges for HuRef gold set variants. GiaB, Illumina platinum genome and Baylor Gold Set (BGS) are shown for comparison. Bin names represent the upper bound in size range.

We defined SVs as insertions or deletions greater than or equal to 50 bp. In total, there were 1,953 insertions and 3,013 deletions in the HuRef SV gold set. This included a wide range of sizes (see Fig. 2). Despite this wide size range, all SVs in the gold set had exact breakpoints, which was a unique feature to this gold set. There was a clear bump at the 256–512 bp bin corresponding to the SINE elements, which was not present in GiaB nor Illumina platinum genome. We also compared to the SV gold set of HS1011 from Baylor College of Medicine^2^. We defined Baylor Gold Set (BGS) as the set of SVs from HS1011 that were supported by long reads. Our HuRef gold set had a comparable number of variants and had the added advantage of including also small variants. Compared to GiaB SVs we have a similar number of deletions and more large insertion. We compared all variants, including the lower confidence ones, for the aforementioned gold sets in Supplementary Figure 1. Despite the relaxed criteria HuRef retained a reasonable variant size distribution that follows closer to the expected exponential or power law decay^13^,^14^ when compared to other gold sets. The slight peaks due to SINE/LINE elements were even more evident.

**Figure 3:**
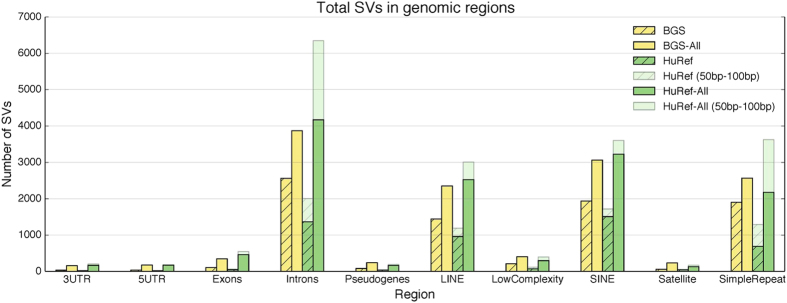
Comparison of HuRef SV counts to Baylor Gold Set (BGS). Suffix of “All” refers to the entire set. We show the variants in the 50–100 bp range separately since BGS defines SVs are >100 bp.

We analyzed the proximity of the SVs in this gold set to classes of repeat elements from RepeatMasker. Some SVs are known to be associated with repetitive elements, such as those mediated by non-allelic homologous recombination (NAHR)^15^. Our analysis confirmed that there was an enrichment of repetitive elements for both deletions and insertions as over 79% of insertion SVs and over 80% of deletion SVs intersected with repeats whereas repetitive elements make up about 50% of the genome . Furthermore, no class of repetitive element was systematically excluded (see Supplementary Table 2). We compared the counts of SVs in genes and repetitive elements in the HuRef gold set to the Baylor Gold Set^2^ (see Fig. 3). The counts were quite similar across all types of elements. The same analysis of repetitive elements was also applied to NSVRs. Again, we did not see any repetitive element that was systematically excluded from NSVRs (see Supplementary Table 3). This ensured that the false positives identified from NSVRs were not greatly biased against these difficult regions.

We analyzed the insertions and deletions detected from the Sanger reads that were not included in the Gold set. Only SVs that were validated by at least two sources were included in the HuRef gold set—we called these the “Gold” SVs. Other SVs that were only validated by one source were called “Pass” and the remaining were “Notval”. We observed that our “Gold” SVs were more enriched with repetitive elements compared to “Pass” and “Notval” (see Supplementary Tables 4 and 5).

### Analysis of small variant gold set

**Figure 4:**
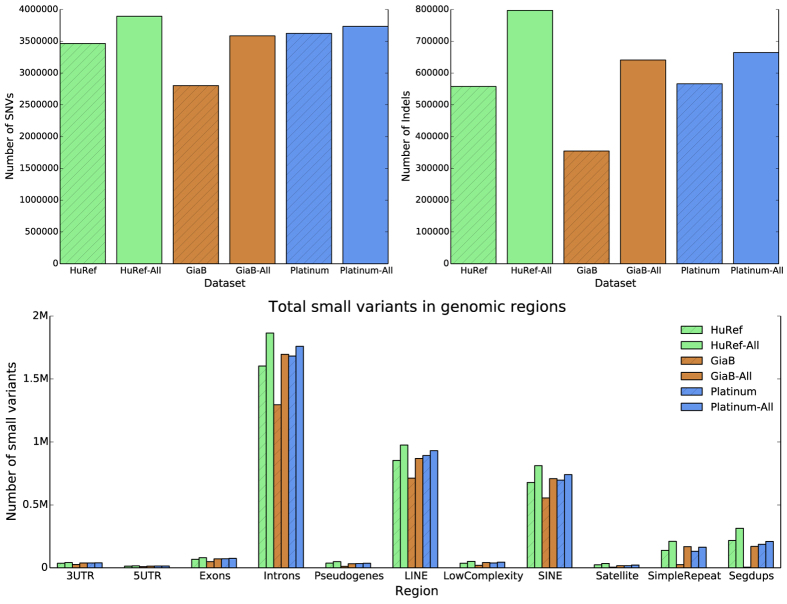
Counts of SNVs and Indels in each gold set. Suffix of “All” refers to the complete set of small variants. The other bar is the gold set variants.

In total, 3,467,077 SNVs and 558,075 Indels were included in the HuRef gold set. Figure 4 shows a comparison of SNVs and Indels counts in three different gold sets. Genome in a bottle (GiaB)^1^, Illumina platinum genome and the HuRef gold set. The HuRef gold set had comparable numbers of variants compared to the Illumina platinum genome even though the platinum genome was built on a trio of individuals. When considering all variants, our gold set had more variants. Also plotted was the count of small variants divided into classes of regions. The HuRef gold set had a comparable number of small variants to Illumina platinum genome, while GiaB was under represented in many repetitive regions. Figure 2 shows the comparison as a histogram of variant size ranges. For almost all the size ranges, the HuRef gold set had more variants. Our gold set is also unique in that it represents a different individual compared to the commonly used NA12878. Furthermore, NS12911 is a male genome, which has variants in non-PAR regions on the Y chromosome. In total there were 2,319 SNVs and 354 Indels in such regions for the HuRef gold set.

As further validation of the gold set, we analyzed the distribution of SNVs and Indels for each gold set which were present in different classes of genomic regions. These regions included genes and repetitive elements. Notably, the HuRef gold set was not absent in any of these classes of regions and had a similar overall distribution compared to the other two gold sets (see Supplementary Figures 2 and 3).

### Application of gold set

The availability of a ground truth for structural variation allows us to compare the performance of several popular SV detection algorithms at a whole genome scale on real data. We compared LUMPY^16^, DELLY^17^, MetaSV^18^, Pindel^19^, BreakDancer^20^, CNVnator^21^ and BreakSeq2^22^. Table 1 compares all tools for deletion SVs. We only considered SVs greater or equal to 100 bp since both LUMPY and DELLY were limited to that range. We were also careful to remove Venter breakpoints from the breakpoint library of BreakSeq2. *F*_1_ score, which is the harmonic mean of precision and recall, was used as the metric for comparison. There were 1,963 deletions in this size range. MetaSV achieved the best *F*_1_ scores, and was also the most precise among all methods. The vast majority of false positives for LUMPY, DELLY and BreakDancer were for SVs less than 800 bp.

## Discussion

We have presented the most comprehensive gold set of variants available for a personal male genome. Compared to current gold sets of the NA12878 genome, our gold set was shown to have a much wider range of variant types and sizes. Unlike HS1011, it encompasses all major classes of variants, from millions of small variants to thousands of high-quality structural variants of up to a hundred thousand base-pairs. Our HuRef gold set was shown to be a valuable resource to scientists for assessing the accuracy of their sequencing algorithms. Its construction leveraged both long Sanger and short Illumina reads combined with many forms of validation. For structural variants, we validated using junction mapping, paired-end mapping, and population datasets. For small variants we validated across multiple sequencing technologies as well as multiple variant-callers. These validation procedures resulted in a more precise set of variants compared to existing HuRef variant sets. Additionally, we identified high-confidence reference regions of the genome which can be used to estimate the false discovery rate of variant callers. We showed that our methodologies did not bias the gold set against any families of repeat elements. Finally, we demonstrated using the HuRef gold set is valuable for comparing structural variant detection algorithms. We envision that the HuRef gold set will be a very valuable resource for the genomics community to perform realistic benchmarking on a wide range of variant calling methods for improved downstream analyses and interpretation. We have released all reads and variants to public repositories. These resources will be updated as new data become available.

## Additional Information

**How to cite this article**: Mu, J. C. *et al.* Leveraging long read sequencing from a single individual to provide a comprehensive resource for benchmarking variant calling methods. *Sci. Rep.* **5**, 14493; doi: 10.1038/srep14493 (2015).

## Supplementary Material

Supplementary Information

## Figures and Tables

**Table 1:** Deletion SVs detection comparison.

Method	TP	TPR	NSVR-FP	NSVR-FDR	*F*_1_ Score
LUMPY	1,671	0.8512	387	0.1880	0.8311
DELLY	1,507	0.7677	360	0.1928	0.7869
MetaSV	1,683	0.8574	32	0.0186	0.9152
Pindel	1,638	0.8344	135	0.0761	0.8769
BreakDancer	1,741	0.8869	6,534	0.7896	0.3401
CNVnator	700	0.3566	82	0.1049	0.5100
BreakSeq2	1,504	0.7662	23	0.0151	0.8619
