# Supplementary material for: Leveraging long read sequencing from a single individual to provide a comprehensive resource for benchmarking variant calling methods
Source: Sci Rep. 2015 Sep 28;5:14493. doi: 10.1038/srep14493 (PMC4585973; doi:10.1038/srep14493)
Supplement: Supplementary Information [file srep14493-s1.pdf]

Leveraging long read sequencing from a single individual to provide a comprehensive resource for benchmarking variant calling methods – supplementary

John C. Mu[1,*], Pegah Tootoonchi Afshar[2,*], Marghoob Mohiyuddin[1,*], Xi Chen[3], Narges Bani Asadi[1], Mark B. Gerstein[4], Wing H. Wong[3,5,†], Hugo Y. K. Lam[1,†]

August 5, 2015

1. Bina Technologies, Roche Sequencing, Redwood City, CA 94065, USA
2. Department of Electrical Engineering, Stanford University, Stanford, CA 94305, USA
3. Department of Statistics, Stanford University, Stanford, CA 94305, USA
4. Program in Computational Biology and Bioinformatics, Yale University, New Haven, CT 06520, USA
5. Department of Health Research and Policy, Stanford University, Stanford, CA 94305, USA

*Authors contributed equally †To whom correspondence should be addressed

1 Supplementary methods

1.1 Datasets used for construction

The following three sequencing datasets were used to build the HuRef gold set:

- Original Sanger reads from [1], 11,541,781 paired-end reads and 8,859,230 single-end reads totaling 27,967,512,620 bases (9× coverage)
- 100× coverage 2×100 bp Illumina sequencing, 150 bp mean insert size, 1,607,313,294 paired-end reads
- 40× coverage 2×100 bp Illumina sequencing, 350 bp mean insert size, 640,288,290 paired-end reads

We performed Illumina sequencing of HuRef due to a lack of freely available short read sequences for HuRef. This set of reads is only minimally used in the construction of the gold set (see Sections 1.2.3 and 1.4). Hence, we encourage researchers to use these reads to call variants for validation with our HuRef gold set.

1.2 Structural variant gold set

We constructed the structural variant gold set by first extending the SVs from [2] with additional SVs from split-read analysis of the Sanger reads and then refining the SV set using computational validation. We used newer versions of BLAT [3] and BWA-MEM [4] in combination with hard filters to detect split reads that were indicative of large SVs. Split-read alignments were found by first aligning all the Sanger sequences with both BLAT and BWA-MEM. BLAT alignments were processed with a split read detection tool [5]. Split-reads from BWA-MEM alignments were extracted with a custom script. These split-reads represented evidence for either insertion or deletion SVs, which were additionally filtered to exclude sequences spanning gaps in the reference assembly. The remaining SVs were then merged with the SVs from Pang et al. and this extended set was refined by retaining SVs which were validated using two or more of the following validation criteria:

- At least one Sanger read is concordant with the SV junction breakpoint
- At least two Illumina reads (from the entire 140× set) are concordant with the junction breakpoint
- At least one discordant Sanger read pair supports the SV
- The SV matches a variant in DGV (excluding Venter SVs) with 90% reciprocal overlap (only used for deletions)

The above construction methodology should exhibit minimal bias towards short reads since the extended set is constructed solely from Sanger reads.

1.2.1 Split-read analysis

Split-read alignments from BLAT are generated by first running BLAT with parameters `-fastmode -tileSize=18`. Then the resulting alignments are processed with a split-read analysis tool [5]. The parameters used for the tool were:

```
min-supported reads = 2
min-max-centerness = 10%
calcAlnRatioThreshold = 0.5 (for deletions)
calcAlnRatioThreshold = 0.0 (for insertions)
```

Hard filters were applied to improve the reliability of the BLAT split-read alignments:

- Mate of read, if available, is aligned with correct orientation
- Mate of read, if available, is aligned within 10 Mb on the same chromosome
- The size of longest single split on the read is greater the half the size of the sum of all splits
- Each of its supporting read segments have more than half of its length covering unique regions. `wgEncodeCrgMapabilityAlign100mer` (from UCSC) is used to determine uniqueness for segments greater than 100 bp, `wgEncodeCrgMapabilityAlign36mer` (from UCSC) is used otherwise.

Only split-read alignments passing all filters were accepted.

Split-read alignments were also generated from BWA-MEM alignments. For reads spanning a large SV, BWA-MEM will split the alignment into primary and supplementary alignments. A custom script was used to extract reliable split-reads from such alignments. BWA-MEM is run with parameters `-a -M`. Split-read alignments from BWA-MEM were required to pass the following filters:

- (Deletion only) SVs do not cover an assembly gap in the reference genome
- Split segments are aligned with same orientation
- (Deletion only) Maximum 30 bp of overlap or unaligned sequence at the breakpoint
- (Insertion only) Maximum 30 bp overlap or gap between aligned position of split segments
- Minimum fragment length of 100 bp on each side of split to ensure more reliable alignment
- Mapping quality score of 60
- (Deletion only) Split segments cover more than 80% of read sequence to ensure more reliable end-to-end alignment

Shorter SVs may be aligned as part of the CIGAR string rather than a split-read. These were accepted based on the following filters:

- Minimum aligned read length of 100 bp (excluding the insertion length for detecting insertion SVs)
- Mapping quality score of 60

Similar to BLAT split-reads, some hard filters were applied to the resulting split-read alignments:

- (Deletion only) SVs do not cover an assembly gap in the reference genome
- Length less than or equal to 1 Mbp
- Each of its supporting read segments have more than half of its length covering unique regions – `wgEncodeCrgMapabilityAlign100mer` (from UCSC) is used to determine uniqueness for segments greater than 100bp, `wgEncodeCrgMapabilityAlign36mer` (from UCSC) is used otherwise

Finally, a non-redundant SV dataset was generated by merging breakpoint resolution SV calls from [2] and the intersection of SV calls (>50% reciprocal overlap) from the two split-read methods above with at least two reads supporting. Split-read SVs were added if there was no overlap to previously reported SVs from [2].

1.2.2 Generation of large insertion contents

In order to perform junction mapping for large insertions, known insertion sequences were required. However, only insertions found by assembly comparison in [1, 2] have known insertion sequences.

Thus, to generate additional sequences for the remaining insertion SVs in the HuRef gold set, we took advantage of the assembled HuRef contigs to find the insertion sequences. We aligned flanking sequences (500 bp) on each side of insertion to HuRef and its unplaced contigs. We then extracted the reference sequence contained within the two alignments if the following criteria were satisfied:

- Both flanking sequences are aligned on the same chromosome or unplaced contig
- Flanking sequences are both aligned on the forward strand
- Detected insertion sequence should be the same length as the reported insertion length

Insertions SVs where no associated insertion sequence could be found are removed from SV gold set.

1.2.3 Junction validation of structural variants

Junction validation is the procedure where we look for reads that are consistent with the SV junctions. For instance, if a deletion SV is real, there should be some reads that align across the split with few edits. Similarly, for insertion SVs there are two junctions to verify.

In order to perform junction validation with short reads, a BreakSeq junction library for deletion and insertion SVs were generated using a 100 bp flanking sequence. Breakpoint sequences mappable to hg19 or any other junction sequence are excluded. BreakSeq2 [6, 7] was used to perform the junction mapping.

BreakSeq2 could not be used for junction mapping of Sanger reads since it is not compatible with long reads. Hence, we used a custom script. Firstly, sequences around each SV region are recorded:

- Deletions: Concatenate 1000 bp on each side of deletion SV
- Insertions: Concatenate 1000 bp on each side of insertion SV and insertion sequence

We call this the junction library. Sanger reads were then aligned to the junction library as well as hg19. An alignment was accepted if it passes all of the following filters:

- Covered at least 50 bp on each side of the junction
- Greater than 80% of read was aligned
- Less than 10% edit distance
- Total length of indels in 10 bp window around SV junction point <2% of SV length. This is to allow for possible novel sequences near the SV junction.

1.2.4 Validation of structural variants via paired Sanger reads

The median and median absolute deviation (MAD) are estimated based on the fragment-size distribution of reliable paired alignments for each Sanger sequencing library. A reliable paired alignment satisfies the following:

- Read and its mate are aligned on the same chromosome with consistent strand
- Mapping quality of 60
- At least 80% of read is aligned
- Aligned sequence is at least 100 bp

We estimate the standard deviation as $1.4826 \times MAD$. A paired alignment is discordant if its insert size more than three standard deviations away from the median.

1.2.5 Validation via DGV

DGV [8] is not used to validate insertions since most of the insertions in DGV do not have exact breakpoints and sizes. For deletions, a 90% reciprocal overlap is required for validation. We removed all SVs reported by [1, 2] from DGV.

1.3 No structural variant region

In order to assess the accuracy of SV-calling, we identified regions of the genome unlikely to have a true SV. These are regions with high coverage of trimmed Sanger reads showing no evidence of SV. Reads were trimmed with an implementation of PHRED's [9] trimming algorithm with PHRED score cut-off of 20. We call these the "no structural variant regions" (NSVR). A location in the NSVR must satisfy the following criteria:

- Covered by at least 5 Sanger reads

- No soft clipping within 50 bp window around location for each read
- Less than 3 edits in the 50 bp window around the location for each read
- Mapping quality of each read greater than or equal to 20 bp

The total size of all NSVR is 1,845,629,109 bp representing about 60% of the human genome. NSVR are used to estimate the FDR, we call this the NSVR-FDR. An SV that does not match a true SV is classed as a false positive if both breakpoints lie in an NSVR. Otherwise, it is classed as unknown and excluded from further analysis. These regions represent relatively easy regions in the genome for SV callers. Hence, the NSVR-FDR may not be representative of true FDR. Nevertheless, it is still possible to use NSVR-FDR to compare SV callers. If a caller has a comparatively worse NSVR-FDR it is likely to have a worse true FDR. However, if the NSVR-FDR of two callers are identical, we cannot say much about the true FDR.

1.4 Small variant gold set

Variants in the HuRef small variant gold set were generated by processing reads from the two sequencing technologies (Sanger and Illumina) using three popular tools (GATK's HaplotypeCaller, FreeBayes and SAMtools mpileup). As indicated in the main text, the idea behind this is to obtain a high confidence set of small variants. The following are the detailed steps involved in this process:

1. The reads were aligned using BWA-MEM (version 0.7.5) to Human Genome Reference build 37 with the decoy sequence.
2. The read alignments were processed using the following GATK tools – IndelRealigner to improve the alignments, followed by BaseRecalibrator to improve the base qualities.
3. The recalibrated alignments were processed by each of the three aforementioned small-variant callers. In case of GATK's HaplotypeCaller, variant quality scores were re-calibrated using the VQSR step. The recommended best practices were followed for running the GATK tools. The version 138 of the dbSNP build was used for all the GATK tools.
4. Each variant caller output was left-aligned to make the outputs consistent as much as possible and also enable checking for common variants across tools.
5. GATK's CombineVCFs was used to merge the six sets of variants in order to enable generation of common variant sets. The `-genotypeMergeOptions UNIQUIFY` option was used to ensure the genotype information from each variant set was not lost.
6. The combined variant set was analyzed to select the set of SNVs and indels which called by two different variant callers for multiple sequencing technologies. Each variant was analyzed and variants consistently called across multiple variant sets were included in the gold set – this means that either the variant was not called in a variant set or it had the same type across multiple sets.

Table 1 summarizes the versions and command-line options used for the small variant callers we used.

Tool	Version	Command-line options
HaplotypeCaller	3.1	`-contamination 0 -hets 0.01 -indelHeterozygosity 1.25e-4 --maxReadsInRegionPerSample 1000 -mbq 10 -minReadsPerAlignStart 5 -ploidy 2 -stand_call_conf 30 -stand_emit_conf 0.1 -ActProbThresh 0.002 --gcpHMM 10 -ERCIS 10 -maxAltAlleles 6 --maxNumHaplotypesInPopulation 128 --minDanglingBranchLength 4 --minPruning 2 -numPruningSamples 1 -globalMAPQ 45`
FreeBayes	0.9.18	`-n 0 -E 3 --min-repeat-size 5 --min-repeat-entropy 0 -m 1 -q 0 -R 0 -Y 0 -Q 10 -z 1 -F 0.2 -C 2 -3 0 -G 1 -! 0 --prob-contamination 10e-9 -B 1000 --genotyping-max-banddepth 6 -W 1,3 -D 0.9`
SAMtools	1.1	`-C 0 -d 250 -q 0 -Q 13 -e 20 -F 0.002 -h 100 -L 250 -m 1 -o 40`
bcftools	1.1	`-c -p 0.5 -P 0.001`

Table 1: Versions and command-line options for the tools used for calling small variants–almost all the options are default. Note that SAMtools is used in combination with bcftools for variant-calling.

1.5 Illumina sequencing materials and methods

1.5.1 Genomic DNA

DNA (catalog ID: NS12911, donated by Dr. J. Craig Venter) was purchased from the Coriell Institute for Medical Research (Camden, NJ).

1.5.2 Library Preparation

To fragment DNA, 1 µg of genomic DNA was first mixed with 2 µl of 10× fragmentation buffer and 2 µl of 10× BSA on ice for 5 min before adding 2 µl of NEBNext dsDNA fragmentase (New England BioLabs, Ipswich, MA). The reaction was incubated at 37 °C waterbath for 20 min and was stopped by the addition of 5 µl of 0.5 M EDTA. Fragmented DNA was then purified with 45 µl of AMPure XP beads per manufacturer's instructions (Beckman Coulter, Brea, CA), and the DNA was finally eluted in 50 µl of 10 mM Tris-Acetate (pH 8.0). To increase the diversity of the fragmented DNA, six independent DNA fragmentation reactions were performed in this experiment. The size distribution of DNA fragments from each reaction was analyzed using the Agilent 2100 Bioanalyzer prior to library preparation.

Libraries were constructed using the KAPA Library Preparation Kit (KAPA Biosystems, Wilmington, MA). Briefly, 200 ng of fragmented DNA from each reaction was first end-repaired using KAPA End-repair enzyme and *E. coli* DNA ligase, followed by 3'-dA tailing. Adaptor ligation of the dA-tailed DNA was then carried out using the TruSeq Universal Adapter and the TruSeq Adapter (Index 1) manufactured by Integrated DNA Technologies (Coralville, IA). The sequence for the TruSeq Universal Adapter [1] is

```
5'-AATGATACGGCGACCACCGAGATCTACACTCTTTCCCTACACGACGCTCTTCCGATCT
```

and the sequence for TruSeq Adapter (Index 1) [2] is

```
5'-GATCGGAAGAGCACACGTCTGAACTCCAGTCACATCACGATCTCGTATGCCGTCTTCTGCTTG
```

[1]Oligonucleotide sequences ©2007-2015 Illumina, Inc. All rights reserved.
[2]Oligonucleotide sequences ©2007-2015 Illumina, Inc. All rights reserved.

Before adaptor ligation, equal molar of these two adapters were annealed in a beaker with approximately 400 ml boiling water for 5 min, followed by slow cooling to room temperature. After adaptor ligation, DNA was purified with two rounds of AMPure XP beads and DNA concentration was measured by using the Qubit dsDNA High Sensitivity Assay Kit (Life Technologies, Carlsbad, CA). A total of 50 ng of DNA was then amplified with the KAPA HiFi DNA polymerase for 5 cycles under the following conditions: initial denaturation at 98 °C for 45 s, 5 cycles of denaturing at 98 °C for 15 s, annealing at 60 °C for 30 s, and extension at 72 °C for 45 s, followed by a final extension of 1 min at 72 °C. The TruSeq PCR Primer 1 `AATGATACGGCGACCACCGAGAT` and the TruSeq PCR Primer 2 `CAAGCAGAAGACGGCATACGAGAT` were used in PCR amplification. After PCR, DNA was purified with AMPure XP beads and eluted in 30 µl of 10 mM Tris-Acetate (pH 8.0). Equal amounts of amplicon from 6 independently digested genomic DNA were pool together and were size-selected using 2% agarose E-Gel SizeSelect (Life Technologies). For sequencing, DNA fragments were collected at 350 bp and 500 bp, followed by AMPure XP beads purification.

1.5.3 Illumina Sequencing

Two libraries were sequenced separately with 100 base paired-end reads on an Illumina HiSeq 2000 instrument in rapid run mode. For the 350 bp fragments, a total of 3,214,626,588 reads generated from 5 sequencing runs was pooled together to obtain 100× coverage. For the 500 bp fragments, a total of 1,280,576,580 reads generated from 2 sequencing runs was pooled together to obtain 40× coverage.

2 Supplementary: Size distribution of all variants

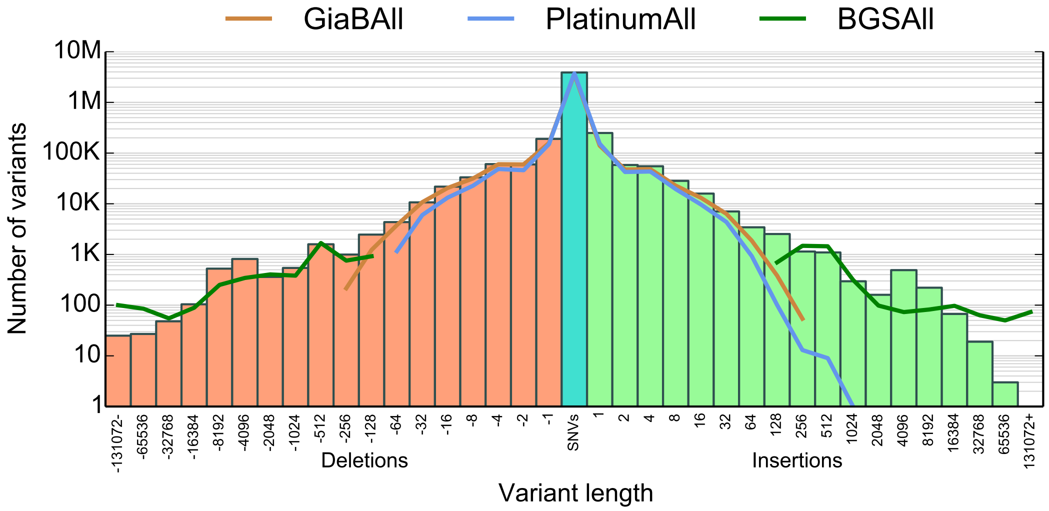

Figure 1: Histogram of size ranges for all HuRef variants. GiaB, Illumina platinum genome and Baylor Gold Set (BGS) are shown for comparison. Bin names represent the upper bound in size range.

3 Supplementary: Repeat analysis of SVs

Repeat type	Count of repeat elements	Count in NSVR	Percent in NSVR
Any repeat	5,399,433	3,910,617	72.43%
LINE	1,550,428	1,200,114	77.41%
SINE	1,782,971	1,272,437	71.37%
Simple repeat	671,754	377,651	56.22%
LTR	740,469	580,056	78.34%
Low complexity	98,488	60,921	61.86%
Retroposon	5,420	3,494	64.46%

Table 2: NSVR intersected with repetitive elements, 50 bp window added to each repeat

Repeat type	Number of insertion SVs	As percentage within all insertion SVs	Number of deletion SVs	As percentage within all deletion SVs	Approximate expected percentage in genome
Any repeat	1,555	79.62%	2,435	80.82%	52.74%
LINE	593	30.36%	861	28.58%	22.77%
SINE	571	29.24%	1,395	46.30%	15.16%
Simple repeat	691	35.38%	741	24.59%	2.15%
LTR	316	16.18%	436	14.47%	9.74%
Low complexity	64	3.28%	85	2.82%	0.35%
Retroposon	15	0.77%	78	2.59%	0.14%

Table 3: Insertion and deletion SVs intersected with repetitive elements, 50 bp window added to each repeat

4 Supplementary: Filtered SVs

Type	All	Gold	Pass	Notval	%Gold	%Pass	%Notval
All	7,917	3,013	2,391	846	38.06%	30.20%	10.69%
Any repeat	5,244	2,435	897	380	46.43%	17.11%	7.25%
LINE	2,191	861	197	45	39.30%	8.99%	2.05%
SINE	2,803	1,395	206	84	49.77%	7.35%	3.00%
Simple repeat	2,098	741	370	183	35.32%	17.64%	8.72%
LTR	1,226	436	122	18	35.56%	9.95%	1.47%
Low complexity	309	85	30	13	27.51%	9.71%	4.21%
Retroposon	241	78	58	64	32.37%	24.07%	26.56%

Table 4: Analysis of filtered deletions

Type	All	Gold	Pass	Notval	%Gold	%Pass	%Notval
All	6,533	1,953	2,885	769	29.89%	44.16%	11.77%
Any repeat	4,449	1,555	1,652	435	34.95%	37.13%	9.78%
LINE	1,692	593	466	78	35.05%	27.54%	4.61%
SINE	1,658	571	423	95	34.44%	25.51%	5.73%
Simple repeat	2,130	691	721	271	32.44%	33.85%	12.72%
LTR	1,004	316	275	42	31.47%	27.39%	4.18%
Low complexity	228	64	45	17	28.07%	19.74%	7.46%
Retroposon	123	15	71	29	12.20%	57.72%	23.58%

Table 5: Analysis of filtered insertions

5 Supplementary: SNV and indel counts in regions

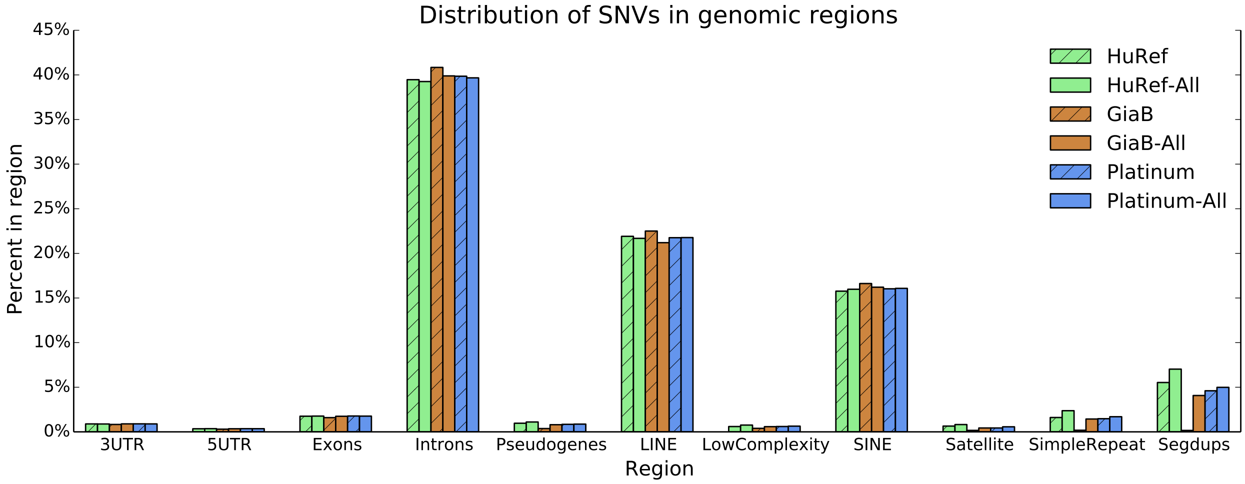

Figure 2: Distribution of SNVs in various genomic regions. Six datasets were compared – the first being our generated gold set, others being subsets of the gold set as well as publicly available gold sets for NA12878. We can observe that the distribution of SNVs in different repeat regions is close to the distribution for gold sets of NA12878.

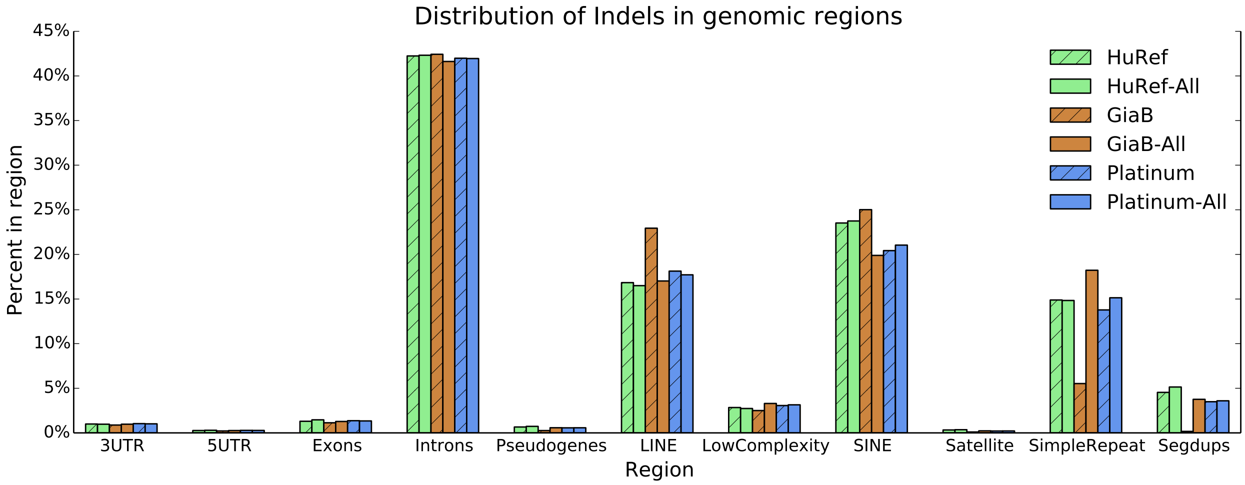

Figure 3: Distribution of indels in various genomic regions. The observed distributions were similar to other publicly available gold sets, just like it was with SNVs.

6 Tandem Repeats Identification

We further enhanced the comprehensiveness of the all call set by identifying the tandem repeats (TRs) in HuRef with the Sanger reads. Using massive sequencing reads to detect genomic TRs is known to be challenging - it is limited by the available read length, as well as the optimization of the current standard mapping tools towards small indels.

We applied a computational method using high-throughput sequencing data for initial identification of TRs, and filtered the results with two whole assembly comparison methods. We chose VNTRseek [10] as the initial detector. Unlike other tools focusing on small TRs with motif size less than 6 nucleotide (nt), VNTRseek can cover motif sizes up to several hundred nt. The tool basically identifies TRs in the reads and the reference genome respectively, and performs comparison between the two sets to identify variations. Because of the sparse Sanger read distribution in some genomic regions, we relaxed the parameter to allow discovering TRs from regions with 1X coverage. At the same time this relaxation may introduce lots of false positives into the results therefore we should carefully filter them out. For validation, we first used Tandem Repeat Finder (TRF) [11] to identify TRs in the HuRef assembly, extended their coordinates for 100bps on each side, and lifted the results to Hg19 using NCBI's Genome Remapping Service (http://www.ncbi.nlm.nih.gov/genome/tools/remap). We defined anything that can be lifted over with coverage between 0.9 and 1.1 to be considered as non-variant, so anything in the VNTRseek results overlapping with this set is potentially a false positive. As a second validation, we identified TRs in the Hg19 again using TRF, extended 100bps from each side, and blat them against HuRef assembly to see whether there are perfect or almost perfect hits, which can be used as a second filter to identify potential false positives in the VNTRseek results. For effective validation, we compared the results from the two filtration methods, and took the intersection as confirmation to filter false positives from the VNTRseek results (Figure 4).

In total we identified 95,852 TRs using the method described above. We successfully identified the previously found uVNTR (upstream variable number of tandem repeats) in HuRef's MAOA gene [1]. As a negative control, we confirmed in our results there is no expansion of the polymorphic trinucleotide (CAG)n repeat in the HTT gene which is involved in Huntington disease.

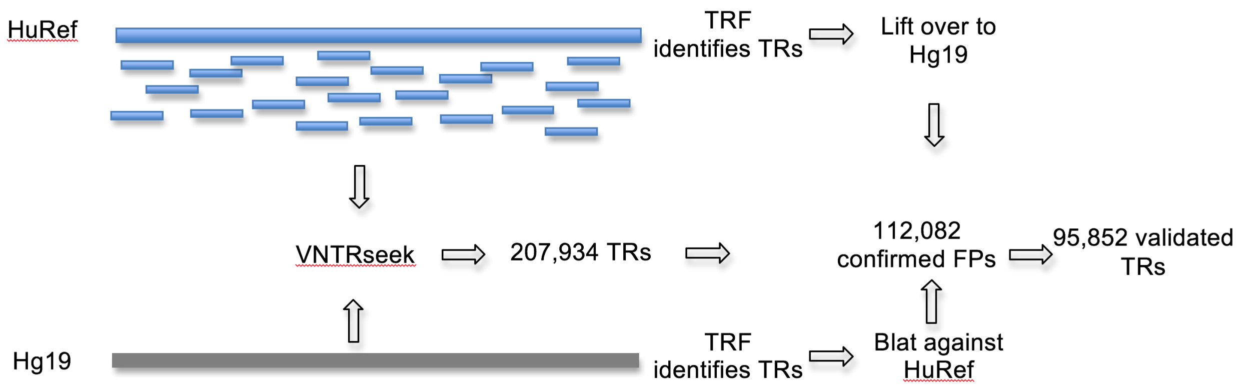

Figure 4: Workflow for constructing the VNTR set

References

[1] Levy, S., Sutton, G., Ng, P. C., Feuk, L., Halpern, A. L., Walenz, B. P., Axelrod, N., Huang, J., Kirkness, E. F., Denisov, G., Lin, Y., MacDonald, J. R., Pang, A. W., Shago, M., Stockwell, T. B., Tsiamouri, A., Bafna, V., Bansal, V., Kravitz, S. A., Busam, D. A., Beeson, K. Y., McIntosh, T. C., Remington, K. A., Abril, J. F., Gill, J., Borman, J., Rogers, Y. H., Frazier, M. E., Scherer, S. W., Strausberg, R. L., and Venter, J. C. Sep 2007 *PLoS Biol.* **5(10)**, e254.

[2] Pang, A. W., MacDonald, J. R., Pinto, D., Wei, J., Rafiq, M. A., Conrad, D. F., Park, H., Hurles, M. E., Lee, C., Venter, J. C., Kirkness, E. F., Levy, S., Feuk, L., and Scherer, S. W. (2010) *Genome Biol.* **11(5)**, R52.

[3] Kent, W. J. mar 2002 *Genome Research* **12(4)**, 656–664.

[4] Li, H. Aligning sequence reads, clone sequences and assembly contigs with bwa-mem (2013).

[5] Zhang, Z. D., Du, J., Lam, H., Abyzov, A., Urban, A. E., Snyder, M., and Gerstein, M. (2011) *BMC Genomics* **12(1)**, 375.

[6] Lam, H. Y. K., Mu, X. J., Stütz, A. M., Tanzer, A., Cayting, P. D., Snyder, M., Kim, P. M., Korbel, J. O., and Gerstein, M. B. dec 2009 *Nat Biotechnol* **28(1)**, 47–55.

[7] Abyzov, A., Li, S., Kim, D. R., Mohiyuddin, M., Stutz, A. M., Parrish, N. F., Mu, X. J., Clark, W., Chen, K., Hurles, M., Korbel, J. O., Lam, H. Y., Lee, C., and Gerstein., M. B. (2015) *Nature Communications* **in press**, –.

[8] MacDonald, J. R., Ziman, R., Yuen, R. K. C., Feuk, L., and Scherer, S. W. (2014) *Nucleic Acids Research* **42(D1)**, D986–D992.

[9] Ewing, B., Hillier, L., Wendl, M. C., and Green, P. mar 1998 *Genome Research* **8(3)**, 175–185.

[10] Gelfand, Y., Hernandez, Y., Loving, J., and Benson, G. jul 2014 *Nucleic Acids Research* **42(14)**, 8884–8894.

[11] Benson, G. jan 1999 *Nucleic Acids Research* **27(2)**, 573–580.
